# Investigating biochemical and structural changes of glycated collagen using multimodal multiphoton imaging, Raman spectroscopy, and atomic force microscopy

**DOI:** 10.1007/s00216-023-04902-5

**Published:** 2023-08-29

**Authors:** Elsie Quansah, Tanveer Ahmed Shaik, Ecehan Çevik, Xinyue Wang, Christiane Höppener, Tobias Meyer-Zedler, Volker Deckert, Michael Schmitt, Jürgen Popp, Christoph Krafft

**Affiliations:** 1 Institute of Physical Chemistry and Abbe Center of Photonics (IPC), Member of the Leibniz Center for Photonics in Infectious Research (LPI), Friedrich Schiller University Jena, Helmholtzweg 4, 07743 Jena, Germany; 2 Leibniz Institute of Photonic Technology (IPHT), Member of Leibniz Health Technologies, Member of the Leibniz Center for Photonics in Infectious Research (LPI), Albert-Einstein-Straße 9, 07745 Jena, Germany

**Keywords:** Collagen crosslinking, Advanced glycation end products, Second harmonic generation, Two-photon excited fluorescence, Raman spectroscopy, Atomic force microscopy

## Abstract

Advanced glycation end products (AGEs) form extracellular crosslinking with collagenous proteins, which contributes to the development of diabetic complications. In this study, AGEs-related pentosidine (PENT) crosslinks-induced structural and biochemical changes are studied using multimodal multiphoton imaging, Raman spectroscopy and atomic force microscopy (AFM). Decellularized equine pericardium (EP) was glycated with four ribose concentrations ranging between 5 and 200 mM and monitored for up to 30 days. Two-photon excited fluorescence (TPEF) and second harmonic generation (SHG) microscopic imaging probed elastin and collagen fibers, respectively. The glycated EP showed a decrease in the SHG intensities associated with loss of non-centrosymmetry of collagen and an increase of TPEF intensities associated with PENT crosslinks upon glycation. TPEF signals from elastin fibers were unaffected. A three-dimensional reconstruction with SHG + TPEF z-stack images visualized the distribution of collagen and elastin within the EP volume matrix. In addition, Raman spectroscopy (RS) detected changes in collagen-related bands and discriminated glycated from untreated EP. Furthermore, AFM scans showed that the roughness increases and the D-unit structure of fibers remained unchanged during glycation. The PENT crosslinked-induced changes are discussed in the context of previous studies of glutaraldehyde- and genipin-induced crosslinking and collagenase-induced digestion of collagen. We conclude that TPEF, SHG, RS, and AFM are effective, label-free, and non-destructive methods to investigate glycated tissues, differentiate crosslinking processes, and characterize general collagen-associated and disease-related changes, in particular by their RS fingerprints.

**Supplementary information:**

The online version contains supplementary material available at 10.1007/s00216-023-04902-5.

## Introduction

Type 1 diabetes is a chronic condition where the body’s immune system destroys the insulin-producing cells, leading to high blood sugar levels [1]. The long-term complications indicate that hyperglycemia is the initiating cause of tissue damage in diabetics [2]. Advanced glycation end products (AGEs) can also be formed by short exposure to glucose, making collagen more susceptible to collagenolysis due to the changes in the equilibrium distribution state [3]. It was hypothesized that hyperglycemia occurs either by stimulation of cellular metabolism, structural and functional changes of tissue proteins, or accumulation of AGEs [4–6]. AGEs are heterogeneous chemical products resulting from nonenzymatic interactions between reducing sugars and proteins known as the Maillard reaction [7, 8]. They form intermolecular crosslinks leading to changes in their physical structure. Elevated AGE levels were observed not only with aging and in diabetics, but also in patients with Alzheimer’s disease, end-stage renal failure, cataracts, and atherosclerosis [9–12].

Pentosidine (PENT) crosslinks are one of the major AGE products formed by nonenzymatic glycation and oxidation of proteins [13]. Over the years, fluorescent crosslinks such as PENT were used as a biomarker for AGEs [14, 15]. Even though only traces of PENT can be discovered in tissue proteins, they are extremely beneficial to analyze cumulative tissue proteins. High levels of PENT are associated with complications of aging and are considered a risk factor and precursor to severe diabetic conditions [16]. Accumulation of PENT was reported to cause alterations to the function and structure of collagen, thus increasing stiffness in cartilage, bones, and skin [2, 17, 18]. The most targeted extracellular matrix (ECM) proteins for glycation are collagen and elastin, which are the structures that provide tensile strength and elasticity [19]. The presence of PENT caused modifications of elastin fibers and collagen fibers, such as stiffening of collagen bundles, thickened fibrils, and loss of elasticity of elastic fibers [20–23]. The conventional method used to investigate AGEs and access the degree of crosslinking is high-performance liquid chromatography (HPLC) [24]. However, this method is time-consuming, requires complex instrumentation, and is a destructive process. Due to these limitations, alternative techniques are necessary. Among these are non-linear imaging techniques combining two-photon excited fluorescence (TPEF) and second harmonic generation (SHG). These techniques allow detecting endogenous fluorophores and collagen in a non-destructive and label-free way. Further, they provide morphochemical information in samples with subcellular spatial resolution [25]. Yazdanfar et al. showed in a type I collagen-rich tissue analysis that TPEF visualizes fluorescence in elastin fibers while SHG imaging detects structures that lack inversion symmetry and are dominated by collagen-rich fibers in the body [26]. Non-linear imaging allows diagnosing diseases (e.g., identifying tumors) via determining the morphological, biomolecular, and biochemical properties of a tissue sample [27–29]. TPEF and SHG were applied to study the deformation of collagen and elastin fibers through the structure and orientation of the fibrils [30]. They were also used to investigate the changes in the morphology of tissues after crosslinking [31–33]. For chemical analysis of scaffolds and tissues during crosslinking, Raman spectroscopy is another well-suited technique used in several studies[34–36]. Likewise, to examine the fibril alignment and mechanical properties of ECM proteins and nanoparticles [37, 38], atomic force microscopy (AFM) was utilized in pericardium samples [39, 40].

As an extension to our previous glutaraldehyde [41] and genipin [36, 42] induced collagen crosslinking research, this work studied ribose as a crosslinker. Despite the wide applicability of glutaraldehyde, it was reported to be toxic to cell survival [43]. Genipin exhibits lower toxicity, yet affects certain cell types [44]. This uncertainty made this crosslinker controversial for researchers. In this case, ribose was employed to investigate faster glycation in comparison to other reducing sugars like fructose and glucose. This significantly reduces the amount of time required for the formation of AGEs. Ribose levels in blood plasma are reported to be in the range of 0.02 mM, fructose in the range of 0.008 to 12 mM, and glucose level in the range of 5 mM. The elevated ribose levels up to 200 mM in our model system should induce effects at a further accelerated time scale. Although concerns were raised about cell viability when administering high doses of ribose, research showed that with low ribose concentrations, cell viability is maintained allowing investigating cell interactions [45, 46]. Therefore, ribose is a cost-effective, facile, and biocompatible crosslinker that has the potential for in vivo investigations with low cytotoxicity.

Our approach employed non-invasive, non-destructive, and label-free techniques such as Raman spectroscopy, SHG, TPEF, and AFM to investigate the biochemical and structural changes of glycated collagen. In vitro analysis of equine pericardium (EP) tissue was performed at three time points (10 days, 20 days, and 30 days). The untreated EP was compared to the glycated EP at four different ribose concentrations of 5 mM, 50 mM, 100 mM, and 200 mM. The fluorescence of PENT crosslinks and elastin fibers was detected in the TPEF channel. The concomitant structural changes in collagen fibers were determined by SHG. To complement the non-linear imaging methods, Raman spectroscopy was utilized to detect the biochemical changes. In addition to optical microscopic imaging techniques, AFM was performed to determine the ultrastructure of untreated and glycated EP. Although considerable research has been done on AGEs, this study is the first to use multiphoton imaging, Raman spectroscopy, and AFM to evaluate PENT crosslinks in EP samples.

## Materials and methods

### Sample preparation

Decellularized EP tissue was provided by Auto Tissue (Berlin, Germany). The tissue was cut into 15 pieces, each having a size of approximately 1 × 1 cm^2^ and thickness of 1 mm. For glycation, the ribose solution was prepared at four different concentrations ranging from 5 mM, 50 mM, 100 mM, and 200 mM in phosphate-buffered saline (PBS) with 44 mM NaHCO_3_ and 25 mM HEPES. Three pieces of tissue were immersed in 2 mL of each ribose concentration, and one tissue sample remained in PBS as a control. The tissues were incubated at 37 °C for 10, 20, and 30 days. All the tissues were washed with PBS at room temperature and kept in PBS during the optical measurements.

### Raman spectroscopy

Raman spectra were acquired using a RXN1 spectrometer equipped with a HoloProbe microscope (Kaiser Optical System, Ann Arbor, MI). A built-in 785 nm multi-mode diode laser (Invictus) was used to illuminate the tissue through a 20 × /NA 0.5 water immersion objective lens (Zeiss, Germany) with an average power of about 130 mW. The source laser was focused on the sample, the Raman signal was collected in backscattering geometry and detected on a Peltier-cooled, back-illuminated, deep-depletion CCD chip (Andor, Belfast, Northern Ireland) after passing through a holographic transmissive grating. The CCD was thermoelectrically cooled to − 60 °C. Raman spectra were acquired with a 4-s exposure time, and a total of 100 spectra as a 10 × 10 grid were collected from each sample.

#### Processing of Raman spectra

The fingerprint region of the Raman spectra from 800 to 1750 cm^−1^ was used for the analysis. The spectra were baseline corrected using an extended multiplicative signal correction (EMSC). A Raman spectrum of untreated EP served as a pure component of the EMSC matrix. The background components include five linear functions. All the Raman spectra were normalized with respect to the intensity of the Raman signal at 1451 cm^–1^, which is assigned to the CH_2_/CH_3_ vibrations of collagen and elastin. The Raman spectral preprocessing was performed using the hyperSpec [47] and cbmodels [48] packages in R.

#### Classification of Raman spectra

The plslda function from the cbmodels [48] package in R was used to perform a partial least-squares − linear discriminant analysis (PLS-LDA) on the combined Raman data for each time point. The combined data sets encompassed 100 spectra each of control EP and EP at 5 mM, 50 mM, 100 mM, and 200 mM ribose, respectively. The performance of PLS-LDA for each time point was evaluated with a *K*-fold cross-validation using a *K* value of 5. For a clearer understanding, the mean of the cross-validation is shown.

### Atomic force microscopy

AFM images of the ribose crosslinked equine pericardium at different time domains of 10, 20, and 30 days were recorded with a Nanowizard 1 Scanning Probe Microscope (JPK Instruments AG, Germany). The AFM measurements were carried out in intermittent contact using TAP 190Al-G cantilever probes (Budget Sensors, Bulgaria). All samples were topographically characterized by recording 2.5 × 2.5 µm^2^ images (each with 512 × 512 pixels) at four different locations.

#### Image analysis using atomic force microscopy

Images obtained from the atomic force microscope were processed with the open-source software Gwydion 2.55 [49]. All topographies were corrected for sample tilts by applying a standard plane and line flattening procedure. In that context, second-order polynomial image analysis was applied to all images. The minimum height of the images was set at 0. Mean square roughness of the whole image was computed from second central moment of data values. The mean square roughness was calculated for four images; overall mean and standard deviation (sd) of four images are presented in the results.

### Nonlinear imaging

A picosecond Ti:sapphire laser (Mira HP, Coherent, Santa Clara, CA, USA) generating 2–3 ps pulses (full width at half maximum) at 832 nm with a repetition rate of 76 MHz was utilized as TPEF and SHG source laser. The imaging system, as previously described in detail [25], will be briefly explained in the following. The illumination beam was coupled into an inverse laser scanning microscope (LSM 510, Zeiss, Jena, Germany) and focused on the tissue sample with a 40 × objective lens (LD C-Apochromat, NA 1.1 W Korr UV–VIS-IR, Zeiss, Germany). The laser power was kept constant throughout the measurements. SHG and TPEF signals were detected by a photomultiplier tube (PMT, Hamamatsu Photonics, Hamamatsu, Japan) in the backward direction. Signals in the epi direction were detected in succession using a combination of shortpass and bandpass filters (SP 650 nm and BP 415/3, Omega Optical, USA for epi-SHG; SP 650 nm and BP 525∕45 nm, Semrock for epi-TPEF) and a PMT. This illumination and detection wavelength configuration for SHG allows accessing the non-centrosymmetric structures in pericardium tissue, exclusively recording the fibrous collagen network, and that for TPEF efficiently images the elastin fibers. Each sample was imaged twice with the same imaging parameters of 2048 × 2048 pixels resolution, 1.6 μs pixel dwell time, and 16 frame averaging. An area scan of 4 × 4 tiles (900 × 900 μm^2^) was obtained for the untreated TPEF/SHG images and one tile (size of 225 × 225 μm^2^) for the composite TPEF/SHG images of the glycation measurements (i.e., the comparison between the untreated EP and the glycated EP at different time points). For the z-stack measurements, images of size 225 × 225 μm^2^ were acquired at a resolution of 1024 × 1024 pixels, a pixel dwell time of 1.6 μs, and a line average of 4. A z-stack depth of 50 μm was measured for the untreated EP and 40 μm for the glycated EP at a z-step size of 0.5 μm. The difference in thickness between the two tissues was due to noticeable elastin fibers in the untreated EP even at the deeper layers (> 50 µm), compared to the glycated EP. To avoid photobleaching, the average laser power was optimized to 30 mW at the surface of the sample, which is below the photodamage threshold [25, 27].

### Analysis of TPEF and SHG images

To quantify the gain of the fluorescence signal and the changes in the SHG signal upon PENT crosslinking, image J software [50] was used to calculate the mean TPEF and SHG intensities (*I*_TPEF_ and *I*_SHG_) of the ECM. On all three glycation days, 15 tissue sections were measured. One untreated sample for each of the time points and four PENT crosslinked EP per glycation day were measured. A total of 30 images were analyzed for both TPEF and SHG ratios. For every image, 10 different regions of interest (ROIs) with a size of 100 × 100 pixels were randomly selected and averaged. The mean TPEF and SHG ratios were calculated according to Eqs. (1) and (2). These two equations demonstrate the changes in the TPEF and SHG channels after PENT crosslinking.

1 $$\mathrm{TPEF}\;\mathrm{ratio}=\frac{I_{TPEF}}{I_{SHG}+I_{TPEF}}$$

2 $$\mathrm{SHG}\;\mathrm{ratio}=\frac{I_{SHG}}{I_{SHG}+I_{TPEF}}$$

## Results

### TPEF and SHG imaging of pericardium tissue glycation

**Fig. 1:**
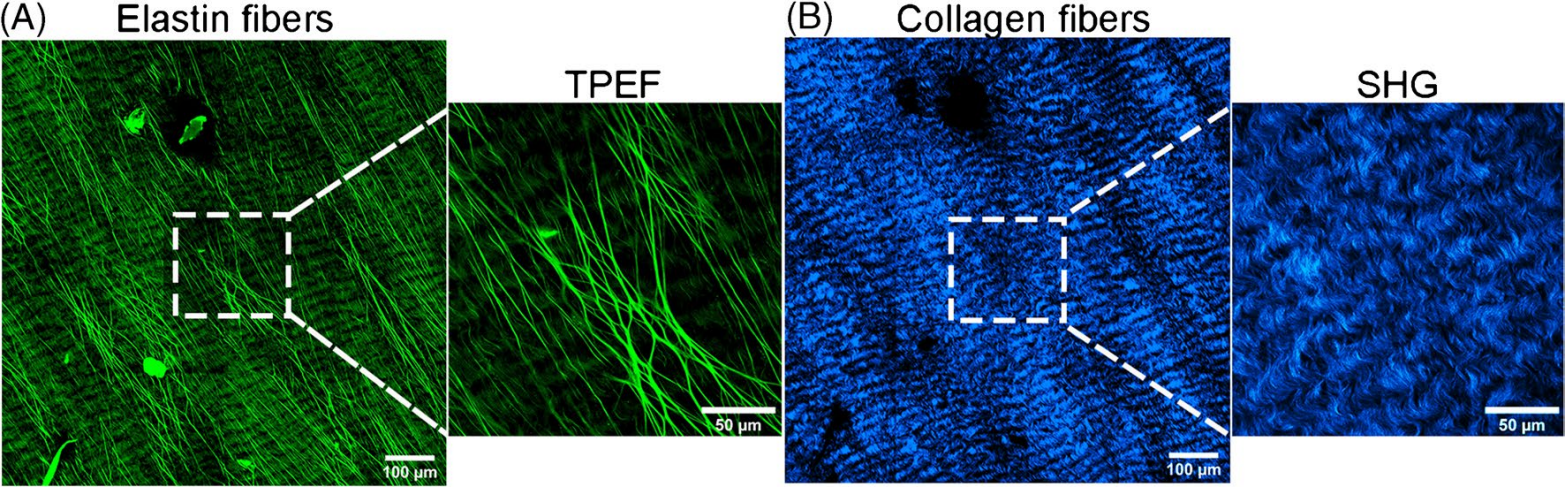
TFEP and SHG images of an untreated EP sample depict the normal morphology of rectilinear elastin (**A**) and wavy collagen fibers (**B**). The dashed squares illustrate a zoomed-in region of interest within the overview images displaying a 4 × 4 tile scan

Our previous studies showed that decellularized EP is composed of collagen and elastin fibers, of which type I collagen is predominately observed in the SHG channel and elastin in the TPEF channel [40, 42]. Figure 1 shows TPEF and SHG overview images. Elastin fibers are parallel and rectilinear arranged and slight branching of individual elastin fibers are identified in the zoomed-in region of interest (Fig. 1A). Since type I collagen is predominant, a strong SHG signal (Fig. 1B) was observed for all untreated EP. The collagen fibers had thin, wave-like structures with well-organized fibrils and a unidirectional arrangement (Fig. 1B). Compared to the previous studies [40, 42], more details are resolved here due to the higher NA of the objective lens (1.1 vs. 0.95), higher sampling rate (2048 × 2048 vs. 512 × 512), higher integration time, and averaging.

**Fig. 2:**
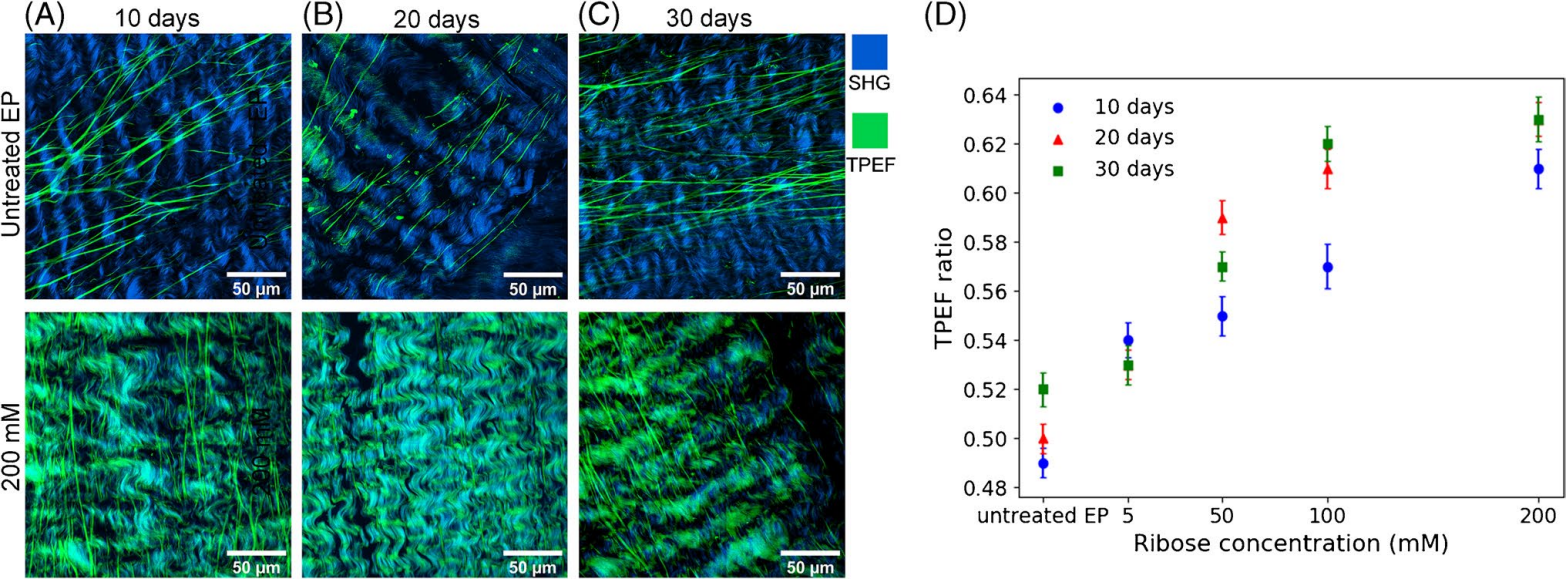
Merged images of TPEF (green) and SHG (blue) comparing the untreated EP and glycated EP at 10 (**A**), 20 (**B**), and 30 (**C**) days of ribose treatment at 200 mM concentration. TPEF ratio against ribose concentration at 10, 20, and 30 days (**D**)

Untreated EP and the glycated EP were analyzed by TPEF and SHG imaging at three different time points (10, 20, and 30 days) for four ribose concentrations to access the structural information from PENT crosslinked EP. The individual TPEF and SHG channels are displayed in the supplementary Figures S1 and S2, respectively. The merged TPEF + SHG images of untreated EP and glycated EP by 200 mM ribose treatment for 10, 20, and 30 days are shown in Fig. 2. The merged TPEF + SHG images of glycated EP by 5 mM, 50 mM, and 100 mM ribose treatment are shown in supplementary Figure S3. Qualitatively, the TPEF intensities increase at higher ribose concentrations which is assigned to increasing glycation of collagen fibers as evident from the green wavy structures. This observation is most dominant at 200 mM ribose concentration. Collagen fibers experience increased crosslinking due to the accumulation of AGEs, which affects the intrinsic fluorescence of collagen. The fluorescent collagen fibers and the concomitant depletion of the SHG signals point to the formation of PENT crosslinks. Furthermore, the TPEF signals from elastin fibers seem to be unaffected by elevated ribose concentrations and increasing glycation, as evident from the fiber morphology, which is similar to untreated EP in Figs. 1A and 2A, B and C.

Based on Eq. (1), the TPEF ratio was calculated for different ribose concentrations. An increase of the mean TPEF ratio is indicative of the formation of PENT crosslinks. This ratio, calculated by averaging 10 ROIs in each image, showed a gradual increase with increasing concentration for 5, 50, 100, and 200 mM concentration (Fig. 2D). The TPEF ratio varied between 0.49 and 0.52 for untreated EP at 10, 20 and 30 days. Variations are also evident for ribose-treated EP with a tendency that the TPEF ratios are higher after 20 and 30 days than after 10 days. 5 mM ribose concentration is an exception where all ratios are similar. Another discrepancy is that 50 mM ribose concentration appeared to have a higher effect for 20 day incubation over 30 day incubation. Possible explanations are that the crosslinks in the native pericardium are not homogenously distributed and/or native crosslinks are present in the particular piece of tissue. An upper TPEF ratio near 0.6 seems to be reached at 100 mM with only a small further increase to 0.63 at 200 mM ribose concentration after 20 and 30 days. Based on Eq. (2), the SHG ratios were calculated. Expected from Eqs. (1) and (2), the SHG ratios decreased in the same way as the TPEF ratios increased (supplementary Figure S4). This intensity decrease is attributed to the alteration of the non-centrosymmetry of collagen fibers upon PENT crosslinking and suggests the formation of new intramolecular and intermolecular PENT crosslinks in the collagen fibers.

**Fig. 3:**
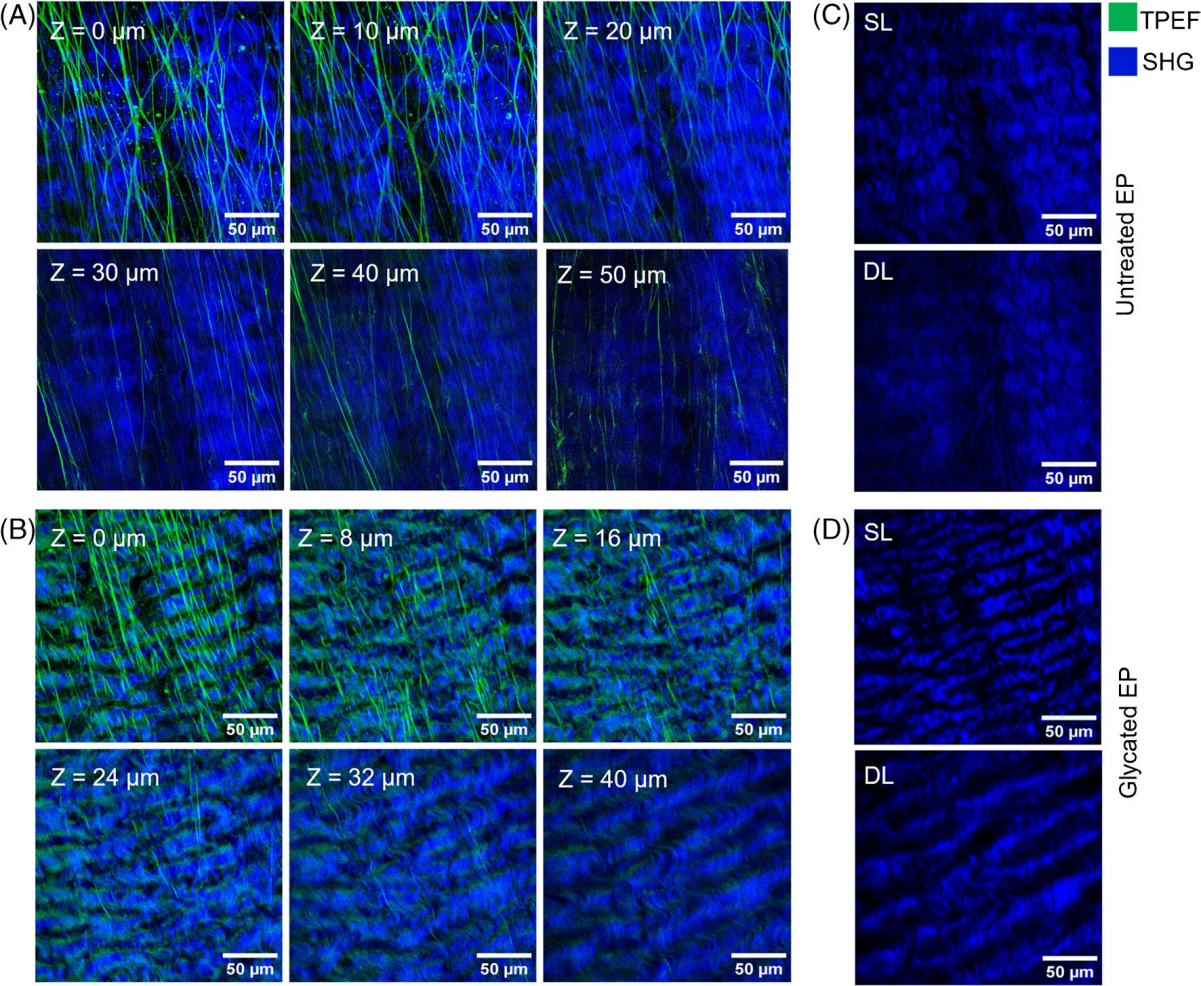
Representation of TPEF and SHG images depicting composite images of z-stack images of the (**A**) untreated EP and (**B**) glycated EP. Elastin fibers (green), detected by the TPEF channel, are abundant in the superficial layers rather than the deeper layers. Almost no TPEF signal was detected in the deep layers. The superficial elastin networks are branched out, but single rectilinear fibers are observed towards the deep layers. The density of fibers was maintained in the (**C**) untreated EP sample, but the glycated EP samples showed expansion of collagen fibers (blue) in the (**D**) deeper layers. Superficial layers: SL, deeper layers: DL

A series of TPEF and SHG images were collected from the surface down to a depth of 50 µm. Z-stack images of merged TPEF + SHG images confirmed the collagen packing in a volume matrix (Fig. 3). SHG images show that the collagen fibers were densely packed and maintained throughout the entire volume of the untreated EP (Fig. 3C), whereas the fibers expanded with increasing depth through the glycated tissue (Fig. 3D). TPEF images show that the superficial elastin networks are branched out, but single rectilinear fibers are observed towards the deep layers. Furthermore, more elastin fibers were found in the superficial layers compared to the deeper layers of the EP tissue where almost no TPEF signal was detected (Fig. 3A and B).

### Raman spectral analysis of pericardium tissue glycation

**Fig. 4:**
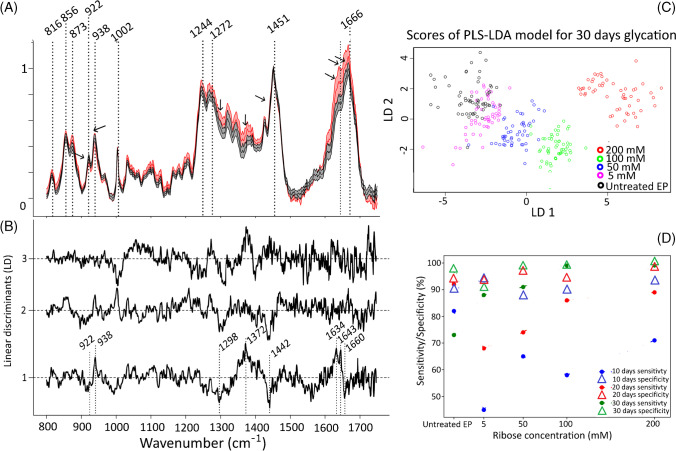
(**A**) Raman spectra of untreated EP and EP after 200 mM tissue glycation after 30 days. The representation of (**B**) loadings and (**C**) LD1 vs LD2 scores of the PLS-LDA model for 30 days of tissue glycation at 5, 50, 100, und 200 mM, (**D**) PLS-LDA performance using *k*-fold cross-validation to classify glycated tissues

The Raman spectra of tissue glycated with 200 mM ribose for 30 days are compared with spectra of untreated tissue in Fig. 4A. Corresponding Raman spectra for 10 and 20 days are shown in Fig. S6. Untreated and glycated tissue show strong Raman bands at 816 cm^−1^ (proline), 856 and 873 cm^−1^ (both hydroxy-proline), 922 and 938 cm^−1^ (both C–C_α_ stretch), 1002 cm^−1^ (phenylalanine), 1244 and 1272 cm^−1^ (both amide III), 1451 cm^−1^ (CH_2_/CH_3_ collagen and elastin), 1640 cm^−1^ (water), and 1666 cm^−1^ (amide I), which can be assigned to type I collagen bands following our previous publication [40]. Unlike the crosslinker Raman bands of genipin [36, 42] and glutaraldehyde [41] observed by Shaik et al., no significant new Raman bands of PENT crosslinks were observed here after the glycation process using ribose. The amide III bands from 1240 to 1320 cm^−1^ and the amide I bands from 1630 to 1670 cm^−1^ tend to be more intense in glycated spectra after normalization to the CH_2/3_ deformation band centered at 1451 cm^−1^. However, background slopes and hydration effects due to the overlapping water band near 1640 cm^−1^ might also contribute to these variations.

The Raman spectral changes were further investigated by PLS-LDA to differentiate and understand the glycation effects on collagen tissue at different time points and ribose concentrations. PLS-LDA was performed for each time point to classify the glycated tissue and identify the associated spectral variations. Figure 4B shows the linear discriminant loadings for the 30 days glycation time point. The LD1 loadings are positive at 938, 1372, 1634, and 1643 cm^−1^, and negative at 922, 1298, 1442, and 1660 cm^−1^. Whereas the loading bands at 922, 938, 1634, and 1643 cm^−1^ are associated with secondary structure changes, bands near 1298 and 1442 cm^−1^ are typical for CH_2_ deformation vibrations of amino acids side chains. As the broad band at 1372 cm^−1^ is neither typical for protein backbone nor amino acids side chains, it may point to ribose and/or crosslinks. The LD1 loading bands are highlighted by arrows in the Raman spectra of 30 days of glycated EP (Fig. 4A). LD2 and LD3 loadings are weaker.

The LD1 vs. LD2 (Fig. 4C) and LD1 vs. LD3 (Fig. S7) score plots show a slight overlap for untreated and 5 mM glycated EP and provide a clear separation for the 50 to 200 mM glycated tissues. The main separation was achieved by LD1. LD2 and LD3 only marginally contributed to the discrimination. Since we did not explicitly observe at PENT Raman band at 1495 cm^−1^ as mentioned in Rubin et al. [51], the changes observed in LDs, in particular LD1, are mainly attributed to the structural changes in collagen resulting from the crosslinking process. A putative crosslinking band was found near 1370 cm^−1^.

Sensitivities and specificities of the PLS-LDA model using five-fold cross-validation are summarized in Fig. 4D. The values indicate how well Raman spectroscopy can discriminate the glycation effect depending on concentration and time. The sensitivity of the untreated EP ranged between 72 and 92%, whereas sensitivity of glycated EP increased as a function of time and ribose concentration. The PLS-LDA model for 10 days has an accuracy of 65%. The misclassifications were largely observed among the untreated EP, the 5 mM, 50 mM, and 100 mM because the Raman spectra for these concentrations showed only minor differences. For 20 days, the accuracy of the PLS-LDA model increased to 82%, and correspondingly, the sensitivity and specificity increased as well. For 30 days, the PLS-LDA could classify the glycated tissue with an accuracy of 90%. At 100 mM and 200 mM ribose concentrations, the sensitivity and specificity were almost 100%. These results are consistent with the largest changes in TPEF and SHG ratios in Fig. 2D and supplementary Figure S4, respectively.

### AFM analysis of pericardium tissue glycation

**Fig. 5:**
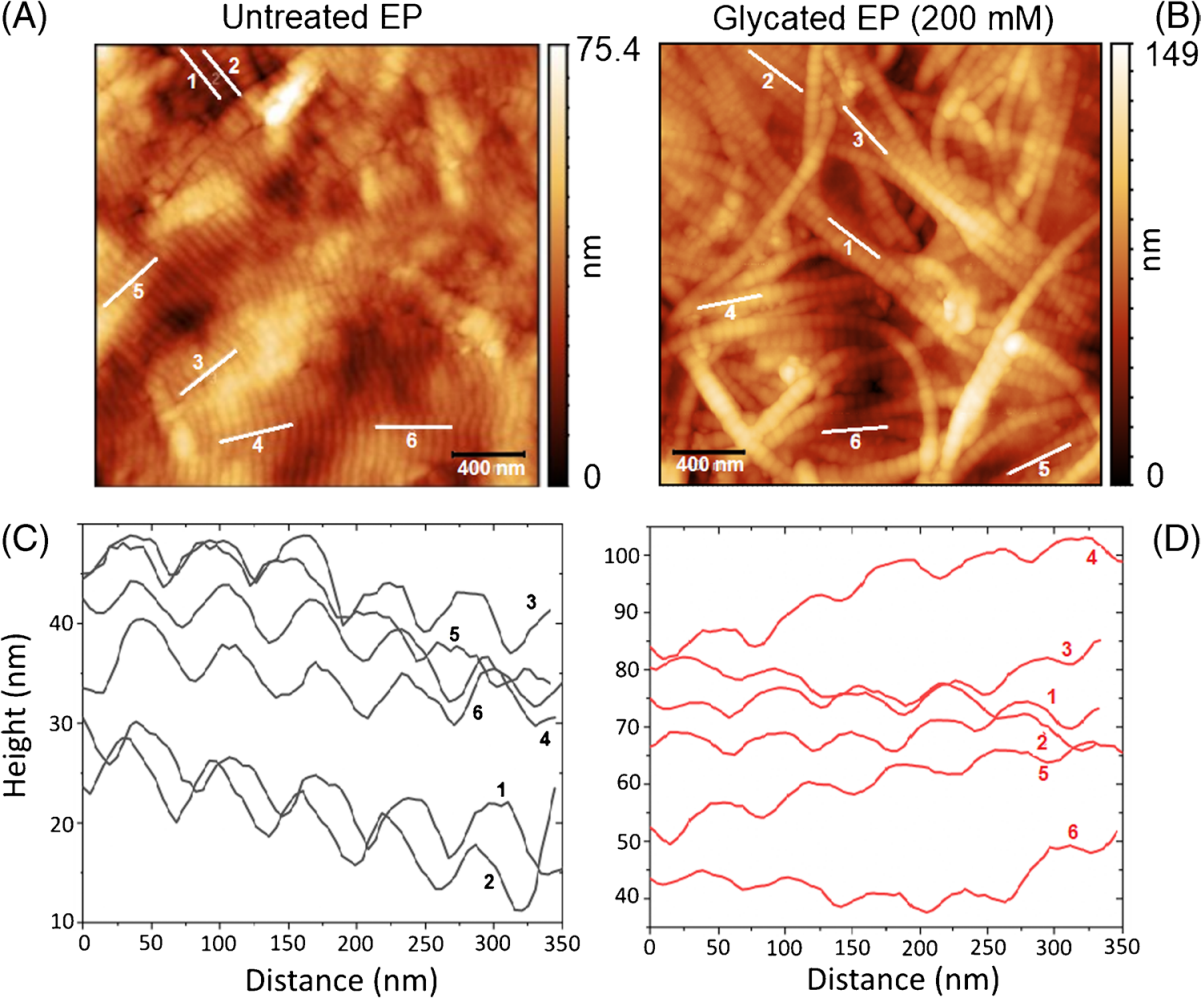
Representative atomic force microscopy topography images of (**A**) the untreated EP and (**B**) the 30 days crosslinked EP with 200 mM ribose, corresponding line profiles of lines 1–6 shown in (**C**) untreated and (**D**) glycated EP

The collected AFM scans of the untreated EP and 30 days of glycated EP at 200 mM ribose concentration are presented in Fig. 5A and B revealing nanostructures of the collagen fibers in the tissue. Mean square roughness was calculated for untreated and glycated EP. Untreated EP has a mean square roughness of 11.64 nm (sd 1.67 nm) and increased to 21.78 nm (sd 1.26 nm) for 200 mM glycated EP.

Based on AFM topographic scans, six lines were chosen along the fibers of untreated EP and 200 mM glycated EP, as shown in Fig. 5A and B. The line profiles in Fig. 5C and D indicate a well-established repeating pattern of collagen, which is commonly denoted as D-unit. The calculated mean length of a single D-unit is 63.8 nm for untreated EP and 64.1 nm for glycated EP. The D-unit lengths agree well with the expected D-period of ≈63 nm and experimental results of 56 to 67 nm in our previous study [42]. However, it is important to note that the length of the D-units in the topography images might be affected by the effective tip diameter. Therefore, the distance between maximum heights of two adjoining crests in line profiles was chosen as the length of D-unit. In summary, the AFM results on collagen fibers show the typical structure in untreated EP and in 30 days of 200 mM ribose crosslinked EP, however with an increased roughness in crosslinked EP compared to untreated EP.

## Discussion

The results of this study are compared in Table 1 with previous research on crosslinking and digestion of pericardium. The glycation crosslinking was the slowest reaction. The pericardium samples were monitored up to 30 days. Genipin and glutaraldehyde crosslinking and collagenase induced digestion already occurred in hours. SHG image contrast of collagen clearly decreased in genipin crosslinking and enzymatic digestion and sensitively probed the loss of non-centrosymmetry and the digestion of collagen fibers. Here, the image contrast is almost unchanged for glycated collagen, and only the SHG ratio decreases which can also be due to TPEF intensity increase. The increase of TPEF was also a sensitive crosslinking marker. In case of glycation, more intense TPEF images were assigned to fluorescent PENT crosslinks. Previously, the analysis of TPEF intensity change accompanying the glycation process showed that collagen is more responsive to the formation of fluorescent AGE than elastic fibers [33]. Increased crosslinking of the collagen fibers due to increasing ribose concentration was also monitored by fluorescence [52, 53]. These results are in agreement with another study, which found that ribose crosslinking increased the TPEF signal in collagen fibers when detected at 525 nm [54]. The increased TPEF intensity after genipin crosslinking was attributed to the formation of a blue pigment with a fluorescence maximum near 625 nm and shift of the fluorescence lifetime to 0.5 ns. Shifting of fluorescence to shorter lifetimes was also observed for glutaraldehyde crosslinking and digestion, however, to a lesser extent.

**Table 1:** Comparison of crosslinking reactions in terms of parameters, SHG microscopy, fluorescence-based techniques, Raman spectroscopy at 785 nm illumination, and other non-optical techniques. Abbreviation: *FLIM* fluorescence lifetime imaging

Reaction	Parameters	SHG	Fluorescence	Raman	Other
Glycation	5–200 mM ribose 10–30 days	Decrease of SHG ratio	TPEF: increase of PENT crosslinks	Increase: 938, 1372, 1634, 1643 cm^−1^. Decrease: 922, 1298, 1442, 1660 cm^−1^	AFM: roughness 11.6 → 21.8 nm, D-period length 64 nm unchanged
Genipin (GE) [42]	0.25% GE 0.5–24 h	Decrease of SHG contrast	TPEF increase, FLIM 2.7 → 0.5 ns, 625 nm band of GE crosslinker	GE bands at 1535 and 1718 cm^−1^, band shifts to 920, 1275, 1412 and 1632 cm^−1^	AFM: roughness 7.6 → 66.8 nm, stiffness 4.4 → 18.9 GPa, loss of period structure
Glutaraldehyde (GA) [41]	0.2–0.6% GA 2 h	Not applied	FLIM 5.3 → 4.4 ns	Pyridinium: 1032 cm^−1^. Increase: 1627 cm^−1^. Decrease: 856, 935, 1282, 1682 cm^−1^	HPLC: pyridinium crosslinks from 3.5–12 to 17–54 ng/mg
Digestion [40]	50 µg/mL collagenase 8–32 h	Decrease of SHG contrast	TPEF: increase, FLIM: 3.8 → 3.5 ns	Increase: 1336, 1665 cm^−1^ Decrease: 814, 852, 938, 1242, 1270 cm^−1^	AFM: Young modulus 3.0–8.6 → 0.5 kPa

Raman spectroscopy offers the highest specificity because each reaction provides a specific fingerprint. Unfortunately, the sensitivity of the glycation-induced spectral changes of Raman bands is relatively low. PLS-LDA indicated collagen structural changes by elevated and reduced band intensities; and the most intense change at 1374 cm^−1^ is broad and unassigned yet. More pronounced Raman bands with clear assignments were identified in previous studies for genipin crosslinks at 1535 and 1718 cm^−1^ due to resonance enhanced bands of the genipin pigment together with band shifts [42] and for glutaraldehyde crosslinks at 1032 cm^−1^ together with intensity variations [41]. Characteristic for collagen digestion are the decrease of collagen bands and the relative increase of elastin-associated bands [40].

The AFM topographic measurements revealed no significant change in the D-period length of the collagen structure between the untreated and glycated EP, but the mean roughness increased from 11.6 to 21.8 nm upon glycation. The roughness increases even more from 7.6 to 66.8 nm, the periodicity of the collagen structures was lost, and the stiffness increased for genipin crosslinked pericardium [42]. The gold standard of glutaraldehyde induced pyridinium crosslinks is high-performance liquid chromatography (HPLC) which correlated well with the photonic measurements [41]. AFM indicated a strong decrease of the Young modulus as a marker for elasticity loss in digested EP [40].

The applied modalities complement each other with respect to the spatial resolution and molecular information. AFM probes collagen fibers of treated and untreated EP on the nanostructure level within small field of views (FOVs) of 2.5 × 2.5 µm^2^. Elastin and collagen fibers were resolved by SHG and TPEF imaging in extended FOVs up to 900 × 900 μm^2^ with confocal depth down to 50 µm. Image details and molecular parameters such as non-centrosymmetry, and fluorescence emission and lifetime were sensitive crosslinking markers. Although Raman images can theoretically be collected at similar resolution than multiphoton images, this would be experimentally impracticable due to extremely long collection. Instead, the hyperspectral capability of Raman spectroscopy was utilized to collect small arrays to compensate small local variation, and Raman spectra provided a specific fingerprint of each crosslinking process.

## Conclusions

Raman spectroscopy and nonlinear, multiphoton imaging techniques such as TPEF and SHG allow analyzing label-free, non-invasively PENT crosslinks. AFM was also applied as a complementary imaging modality to detect the collagen structural stability. The results were compared with previous work of our group and with the literature. An increase in the fluorescence signal generated by PENT crosslinks and a corresponding reduction in the second harmonic signal were observed. In addition, the volumetric analysis provided a further view of the arrangement of the ECM throughout the tissue. The elastin fibers were condensed in the superficial layers, but the deeper layers were devoid of fibers. Moreover, the tightly packed collagen network was lost in deeper layers of glycated EP because of crosslinking. Raman spectroscopy powered by multivariate PLS-LDA analysis increased the biochemical specificity of the analysis and was able to classify 20- and 30-day glycated EP with high accuracy. The Raman spectra of glycated EP showed characteristic changes of band intensities, but no direct crosslinker marker bands that were evident after glutaraldehyde and genipin crosslinking. The AFM topographic measurements probed the ultrastructure of collagen upon glycation and the changes were detected in terms of surface roughness on the nanometer level. In contrast to previous studies of genipin crosslinked EP, PENT crosslinking did not lead to changes of the structure of the collagen fibrils (i.e., in the length of the D-units).

Overall, the present study demonstrated the potential of optical imaging methods to monitor glycation and related structural changes in the pericardium tissue. The application of these combined optical and AFM-based techniques provides a comprehensive biochemical characterization as well as label-free imaging of tissues, which makes them a valuable tool for monitoring the effects of glycation on diabetic patients and collagen-associated changes in general which are related to numerous diseases.

### Supplementary Information

Below is the link to the electronic supplementary material.

Supplementary file1 (DOCX 9762 KB)
