# Supplementary material for: Investigating biochemical and structural changes of glycated collagen using multimodal multiphoton imaging, Raman spectroscopy, and atomic force microscopy
Source: Anal Bioanal Chem. 2023 Aug 29;415(25):6257–67. doi: 10.1007/s00216-023-04902-5 (PMC10558391; doi:10.1007/s00216-023-04902-5)
Supplement: Supplementary file 1 — Supplementary file1 (DOCX 9762 KB) [file 216_2023_4902_MOESM1_ESM.docx]

**Supplementary information:**

**Investigating biochemical and structural changes of glycated collagen using multimodal multiphoton imaging, Raman spectroscopy and atomic force microscopy**

Elsie Quansah*^1,2^, Tanveer Ahmed Shaik*^2^, Ecehan Cevik^2+^, Xinyue Wang^1,2+^, Christiane Höppener^1,2^, Tobias Meyer-Zedler^1,2^, Volker Deckert^1,2^, Michael Schmitt^1,2^, Jürgen Popp^1,2^ and Christoph Krafft^2^

^1^ Friedrich Schiller University Jena, Institute of Physical Chemistry and Abbe Center of Photonics (IPC), Member of the Leibniz Center for Photonics in Infectious Research (LPI), Helmholtzweg 4, D-07743 Jena, Germany

^2^ Leibniz Institute of Photonic Technology (IPHT), Member of Leibniz Health Technologies, Member of the Leibniz Center for Photonics in Infectious Research (LPI), Albert-Einstein-Straße 9, D-07745 Jena, Germany

**Equal first-author, ^+^Equal second author, ^#^Corresponding author:* [*Christoph.krafft@leibniz-ipht.de*](mailto:Christoph.krafft@leibniz-ipht.de)


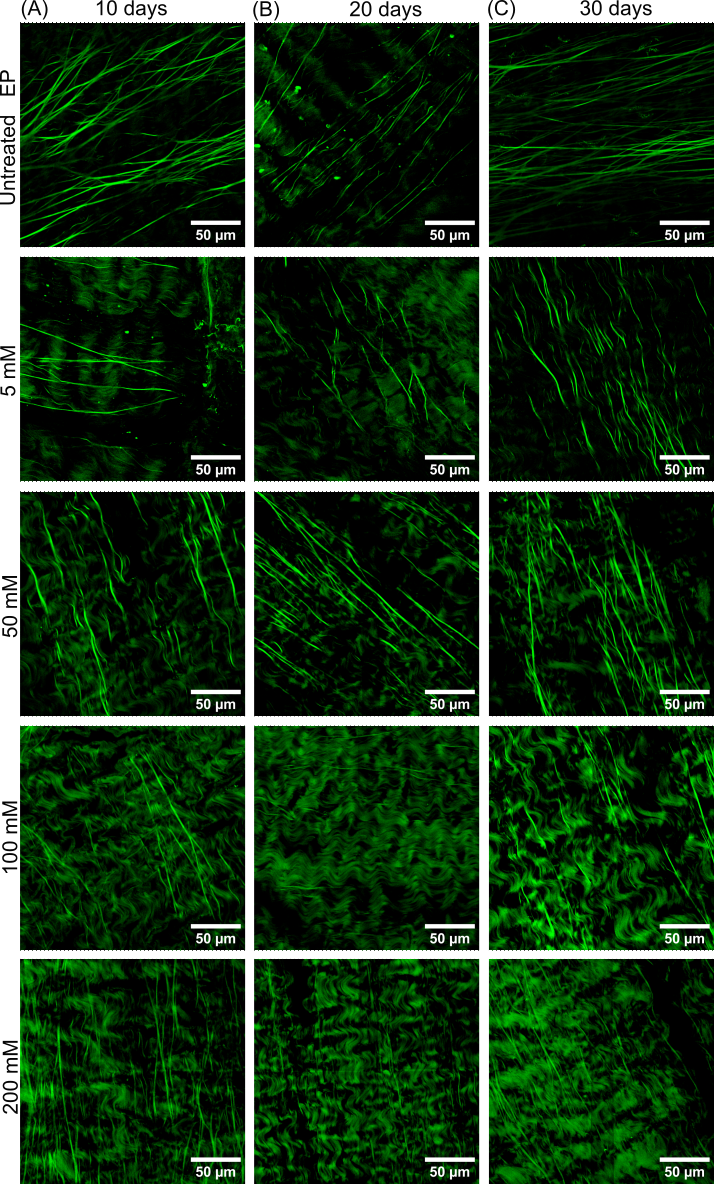


Figure S1: TPEF images of untreated EP and glycated EP at (A) 10 days, (B) 20 days, and (C) 30 days of ribose treatment between 5 and 200 mM.


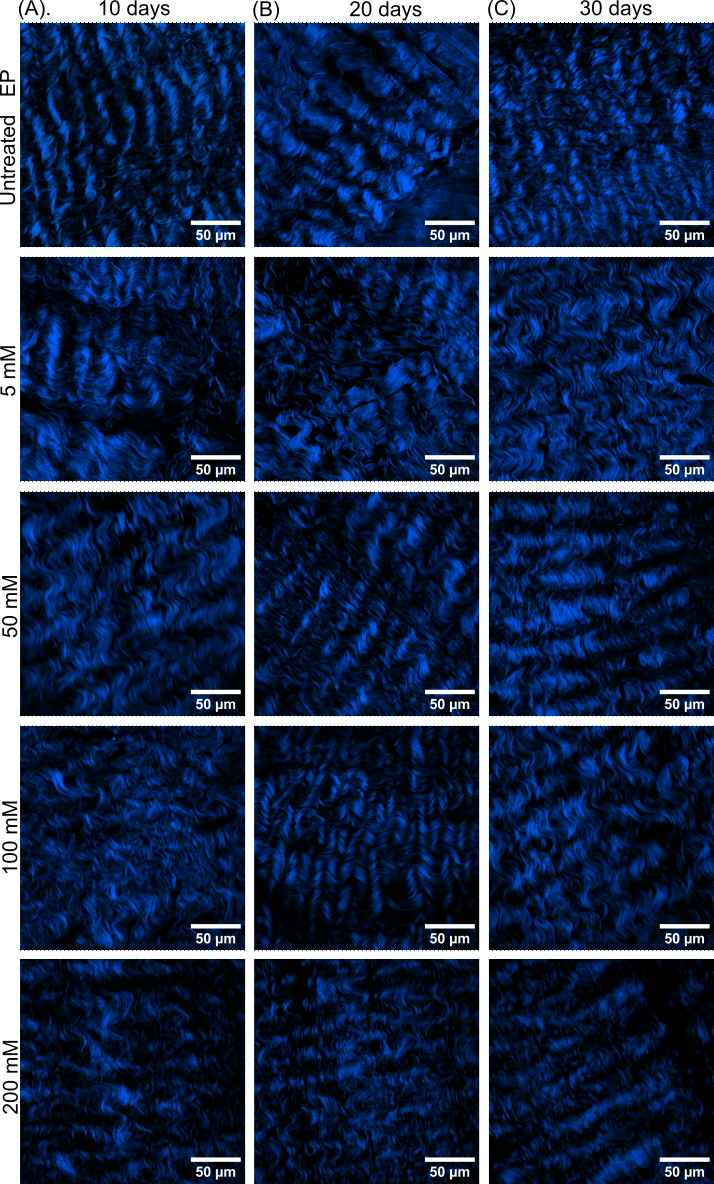


Figure S2: SHG images of untreated EP and glycated EP at (A) 10 days, (B) 20 days, and (C) 30 days of ribose treatment between 5 and 200 mM


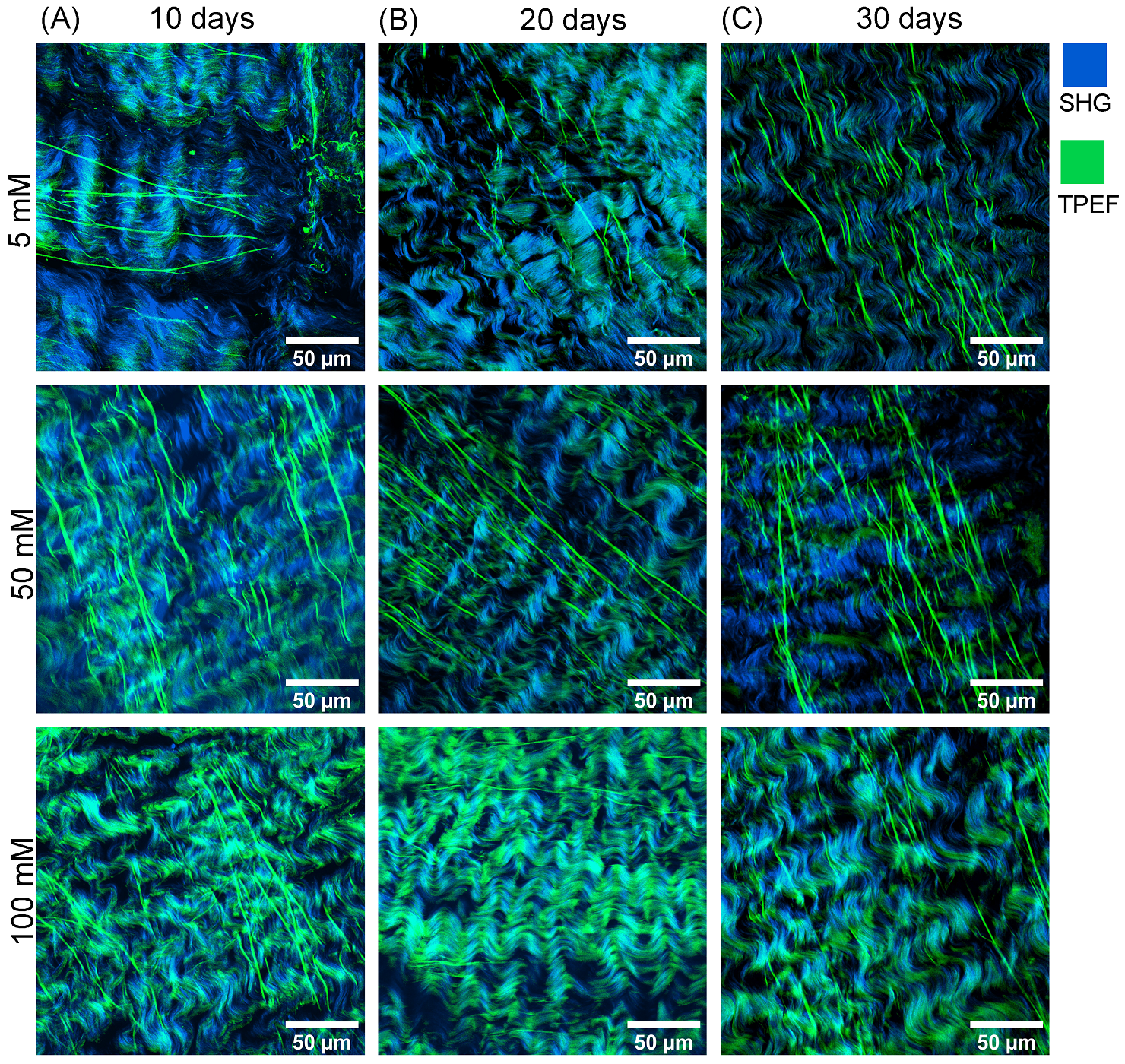


Figure S3: Merged images of TPEF (green) and SHG (blue) of glycated EP at (A) 10 days, (B) 20 days, and (C) 30 days of ribose treatment at 5 mM, 50 mM and 100 mM. The fluorescent signal increases relative to the SHG signal which is assigned mostly to the formation of PENT cross-links and a concomitantly decrease in the SHG signal from collagen.


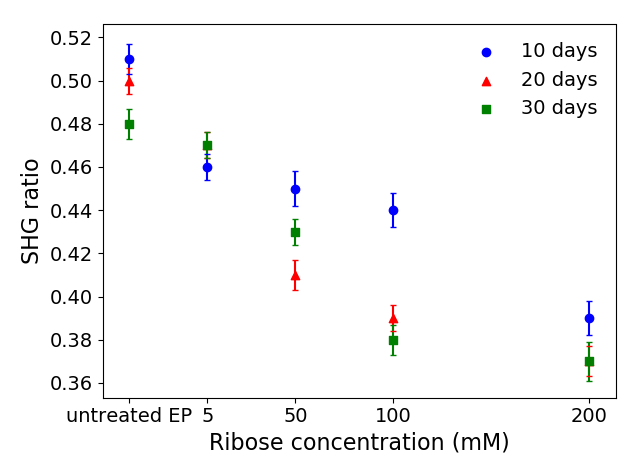


Figure S4: SHG ration against ribose concentration at 10, 20 and 30 days.

**
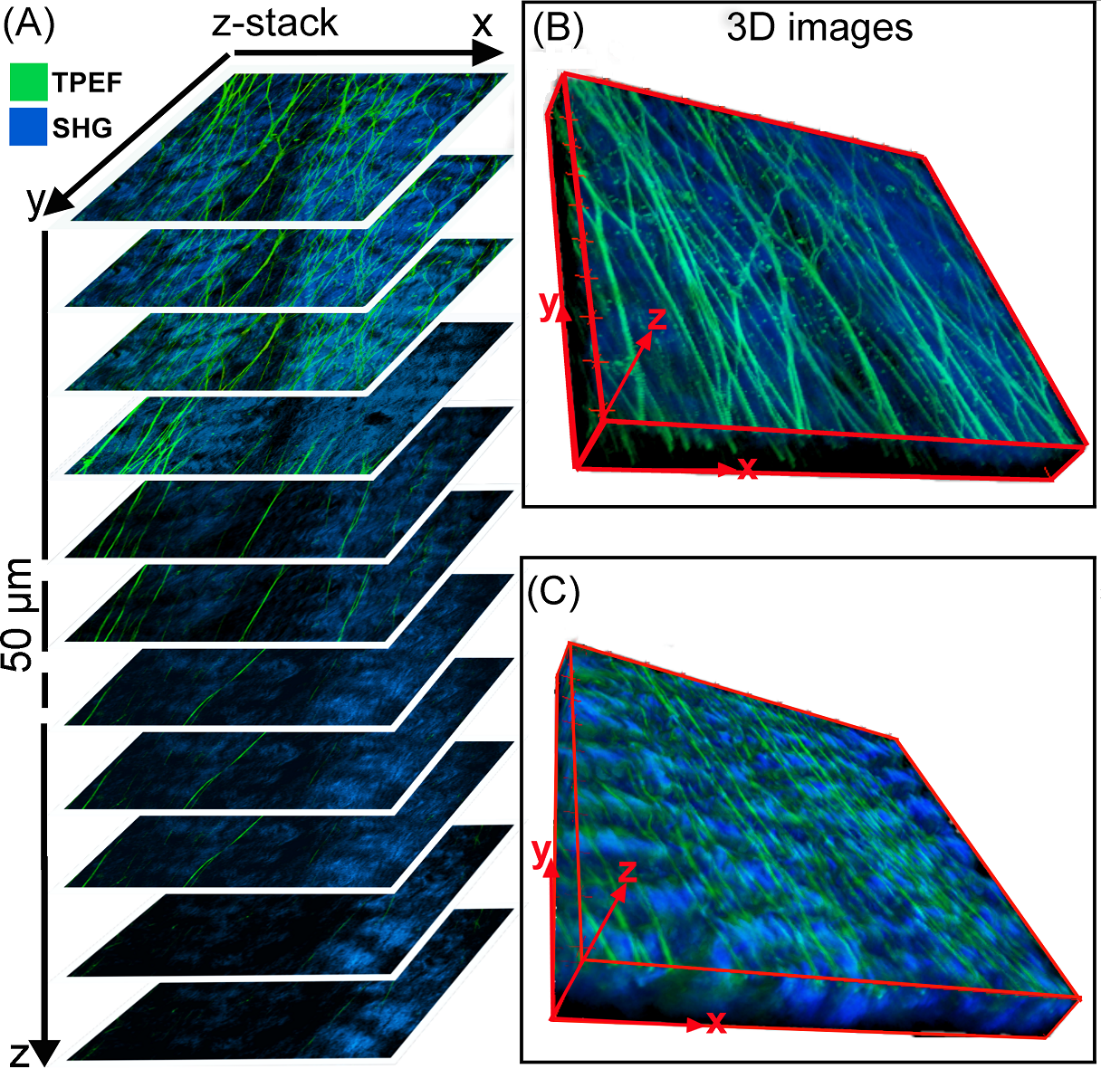
**

Figure S5: Representation of TPEF and SHG images depicting composite images of (A) z-stacks to create a 3D model of the (B) untreated EP and (C) glycated EP. Elastin fibers (green), detected by the TPEF channel, are abundant in the superficial layers rather than the deeper layers. Almost no TPEF signal was detected in the deep layers. The superficial elastin networks are branched out, but single rectilinear fibers are observed towards the deep layers.


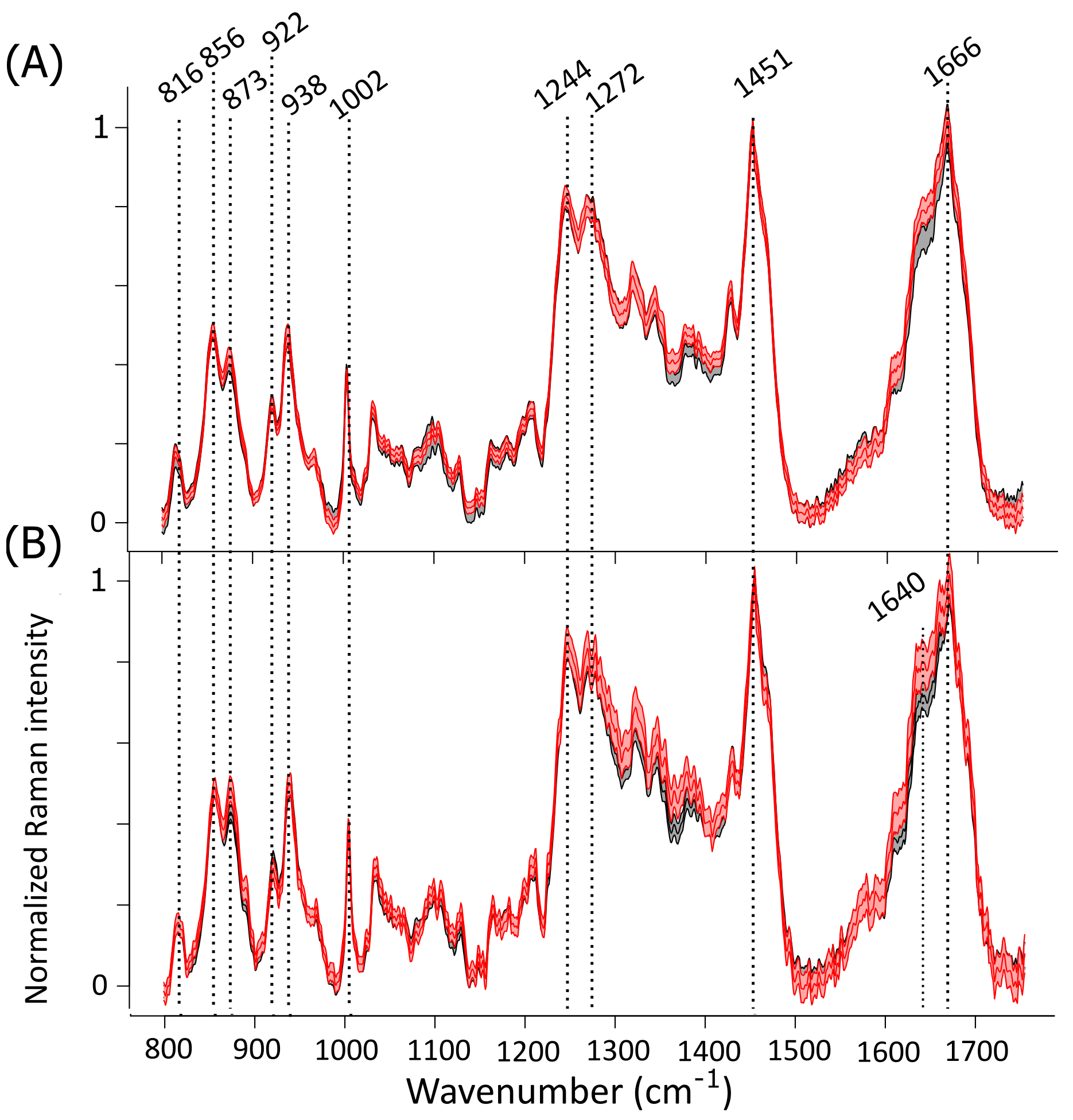


Figure S6: Raman spectra of untreated (black trace) and glycated (red trace) treated with 200 mM ribose for 10 (A) and 20 (B) days.


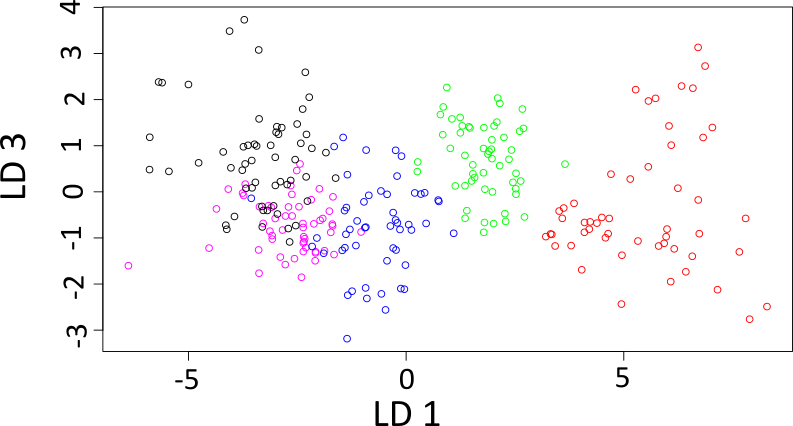


Figure S7: LD1 vs. LD3 scores of the PLS-LDA model for 30 days of tissue glycation.
